# Identification of Prognostic Markers of DNA Damage and Oxidative Stress in Diagnosing Papillary Renal Cell Carcinoma Based on High-Throughput Bioinformatics Screening

**DOI:** 10.1155/2023/4640563

**Published:** 2023-02-04

**Authors:** Le Li, XuKai Liu, Yong Wen, Pan Liu, Ting Sun

**Affiliations:** ^1^Department of Emergency, Zhuzhou Hospital Affiliated to Xiangya School of Medicine, Central South University, Zhuzhou 412007, China; ^2^Department of Neurosurgery, Zhuzhou Hospital Affiliated to Xiangya School of Medicine, Central South University, Zhuzhou 412007, China; ^3^Department of Neurosurgery, The First People's Hospital of Changde City, Changde 415000, China

## Abstract

**Purpose:**

Papillary renal cell carcinoma (pRCC) is the second most common histological subtype of adult kidney tumors, with a poor prognosis due to limited understanding of the disease mechanism. Herein, we have performed high-throughput bioinformatic screening to explore and identify potential biomarkers of DNA damage and oxidative stress for pRCC.

**Methods:**

RNA sequencing data related to pRCC were downloaded from the TCGA database, and differentially expressed genes (DEG) were identified by a wide variety of clustering and classification algorithms, including self-organized maps (SOM), artificial neural networks (ANN), support vector machines (SVM), fuzzy logic, and hyphenated techniques such as neuro-fuzzy networks. Then DAVID and STRING online biological information tools were used to analyze functional enrichment of the regulatory networks of DEG and construct a protein-protein interaction (PPI) network, and then the Cytoscape software was used to identify hub genes. The importance of key genes was assessed by the analysis of the Kaplan–Meier survival curves using the R software. Lastly, we have analyzed the expression of hub genes of DNA damage and oxidative stress (BDKRB1, NMUR2, PMCH, and SAA1) in pRCC tissues and adjacent normal tissues, as well as the relationship between the expression of hub genes in pRCC tissues and pathological characteristics and prognosis of pRCC patients.

**Results:**

A total of 1,992 DEGs for pRCC were identified, with 1,142 upregulated ones and 850 downregulated ones. The DEGs were significantly enriched in activities including DNA damage and oxidative stress, chemical synaptic transmission, an integral component of the membrane, calcium ion binding, and neuroactive ligand-receptor interaction. cytoHubba in the Cytoscape software was used to determine the top 10 hub genes in the PPI network as BDKRB2, NMUR2, NMU, BDKRB1, LPAR5, KNG1, LPAR3, SAA1, MCHR1, PMCH, and NCAPH. Furthermore, the expression level of hub genes BDKRB1, NMUR2, PMCH, and SAA1 in pRCC tissues was significantly higher than that in the adjacent normal tissues. Meanwhile, the expression level of hub genes BDKRB1, NMUR2, PMCH, and SAA1 in pRCC tissues was significantly positively correlated with tumor stage, lymph node metastasis, and the histopathology grade of pRCC. In addition, high expression levels of hub genes BDKRB1, NMUR2, PMCH, and SAA1 were associated with a poor prognosis for patients with pRCC. Univariate and multivariate analyses showed that the expression of hub genes BDKRB1, NMUR2, PMCH, and SAA1 were independent risk factors for the prognosis of patients with pRCC.

**Conclusion:**

The results of this analysis suggested that BDKRB1, NMUR2, PMCH, and SAA1 might be potential prognostic biomarkers and novel therapeutic targets for pRCC.

## 1. Introduction

Renal cell carcinoma (RCC), also known as kidney cancer, is derived from renal tubular epithelial cells and is the most common solid tumor of the kidney, accounting for 3% of adult malignant tumors [1]. It is a heterogeneous group of cancers arising from renal tubular epithelial cells that encompasses 85% of all primary renal neoplasms. Papillary renal cell carcinoma (pRCC) is the second most common histological subtype after clear cell renal cell carcinoma (ccRCC), and 10–15% of RCC histological types are papillary renal cell carcinoma [2]. There are two subtypes of pRCC, type I (basophilic) and type II (acidophilic), and type I has a better prognosis than type II [3]. Most research studies on kidney cancer has focused on ccRCC, and the related studies have shown that compared with ccRCC patients, pRCC patients typically have a lower stage and grade of tumor as well as longer overall survival [4]. The molecular mechanism of pRCC has not been clearly defined. With poor sensitivity to radiotherapy and chemotherapy, surgery is the preferred method for treatment of pRCC, but some patients are prone to metastasis and relapse after surgery. With continued advances in molecular medicine in recent years, the study of the occurrence, development, and metastasis mechanisms of pRCC can help to guide clinical diagnosis and treatment.

The cancer genome atlas (TCGA) project is a joint project of the National Cancer Institute and the National Human Genome Research Institute and aims to apply high-throughput genome analysis technology and to improve the ability to prevent, diagnose, and treat cancer. The cancer genome atlas (TCGA) research network includes analysis of a large number of human tumors to discover molecular aberrations at the DNA, RNA, protein, and epigenetic levels [5]. In this study, TCGA data were used to investigate genes that are deferentially expressed in pRCC. To mine the key genes related to pRCC occurrence and development, we conducted differential gene enrichment (Gene Ontology, GO) analysis and KEGG pathway enrichment analysis, constructed PPI interaction networks, screened hub genes, and performed survival analysis.

## 2. Materials and Methods

### 2.1. Data Collection

The published transcriptome data related to papillary renal cell carcinoma were downloaded from TCGA (https://cancergenome.nih.gov/). The data included 289 papillary renal cell carcinoma samples and 32 normal kidney tissues.

### 2.2. Identification of DEGs

We have performed the edgeR software package in R language (version 3.5.3, https://www.r-project.org/) and a wide variety of clustering and classification algorithms, including self-organized maps (SOM), artificial neural networks (ANN), support vector machines (SVM), fuzzy logic, and hyphenated techniques such as neuro-fuzzy networks to standardize the data and analyze differential expression. Genes with |logFC| > 2.0 and FDR <0.05 were considered differentially expressed genes. To visualize the data graphically, the ggplot2 software package was used.

### 2.3. GO and KEGG Pathway Analysis

The DAVID database (DAVID; https://david.ncifcrf.gov) was used to perform annotation, visualization, and integrated discovery on the genes identified as significantly differently expressed [6]. Using DAVID, GO analysis was performed, including the analysis of cellular components (CC), molecular functions (MF), and biological process (BP) terms. A value of *P*  <  0.05 was considered statistically significant. The Kyoto Encyclopedia of Genes and Genomes (KEGG) (https://www.genome.jp/kegg/) is a knowledge base for systematic analysis of gene functions, linking genomic information with higher order functional information [7]. An adjusted *P* value <0.05 was considered statistically significant.

### 2.4. Hub Genes Selection and Analysis of Modules from PPI Networks

The STRING database (http://string-db.org) aims to provide a critical assessment and integration of protein-protein (PPI) interactions [8]. STRING was used to analyze the selected differentially expressed genes and construct a PPI network. Then, cytoHubba in Cytoscape software (version 3.7.2) was used to screen the top 10 hub genes in the PPI network [9].

### 2.5. Survival Analyses of Hub Genes

The expression profiles and clinical data of 289 pRCC samples were downloaded from TCGA (http://tcga-data.nci.nih.gov) for the survival analysis of hub genes. The Kaplan–Meier method was used for the survival analysis, and log-rank *P* values were calculated. A log-rank *P* value <0.05 was considered statistically significant.

### 2.6. Clinical Specimens

A total of 60 paired pRCC samples and adjacent normal renal specimens were collected from Zhuzhou Central Hospital between June 2016 and June 2021. Inclusion criteria for specimen collection: (1) Postoperative pathology examination confirmed pRCC; (2) the patients with neither radiotherapy nor chemotherapy; (3) complete follow-up data were available; (4) the patients understood the purpose and requirements of the study, agreed to participate in the study, and signed a written informed consent, which was reviewed and approved by the Ethics Committee of Zhuzhou Central Hospital.

### 2.7. Total RNA Isolation and Quantitative Real-Time Polymerase Chain Reaction (qRT-PCR)

The RNA was isolated by TRIzol® reagent (Ambion; USA) from pRCC tissues according to the manufacturer's protocols. And cDNA was reversely transcribed by PrimeScript RT reagent kit (Takara, China). We conducted RT-qPCR on an ABI 7500 RT-PCR system using the SYBR Premix Ex TaqII Kit (Takara, China). All quantifications were normalized to the level of glyceraldehyde phosphate dehydrogenase (GAPDH) in the reaction.

  rimers of
  BDKRB1 was Forward (5′–3′) CAC-TGT-CCT-ACC-GTC-TTT-GTCT,
  Reverse (5′–3′) CGC-AAA-TCT-TGG-TAG-GTG-GT;
  NMUR2 forward (5′–3′) GGC-AAG-GCC-ATG-TGT-AAG-ATC,
  Reverse (5′–3′) GTA-AAA-CGA-CGG-CCAG;
  PMCH forward (5′–3′) CAC-TGT-CCT-GAC-CGT-CTT-TGT-CT,
  Reverse (5′–3′) CCA-TAT-GCC-TGT-GGA-GTG-GAA;
  SAA1 forward (5′–3′) ACC-TGA-GGA-GCC-CCA,
  Reverse (5′–3′) TCT-GCT-CCT-GGC-AGG-CC.

The comparative threshold cycle (CT) method, which compares the differences in CT values between common reference RNA and target gene RNA, was used to obtain the relative fold changes in gene expression. The expressions were calculated by 2^−ΔΔct^ method. Each experiment was performed in triplicate and repeated three times.

### 2.8. Statistical Analysis

SPSS 24.0 software was used for statistical analysis, and GraphPad Prism 7.0 software was used for analysis and mapping. All measurement data in the form of mean ± standard deviation (SD), according to two groups and multiple groups of measuring data comparison using Student's *t*-tests and one-way ANOVA. The relationship between the RNA expression levels of hub genes BDKRB1, NMUR2, PMCH, and SAA1 in the patients with pRCC tissue samples and the clinical pathological characteristics of patients with pRCC was analyzed through Pearson's Chi-squared test, and the relationship between the expression of hub genes BDKRB1, NMUR2, PMCH, and SAA1 and the prognosis of pRCC patients was analyzed by Kaplan–Meier survival analysis and the Cox proportional hazard model. *P*  <  0.05 was considered to be significantly different.

## 3. Results

### 3.1. Identification of DEGs

**Figure 1:**
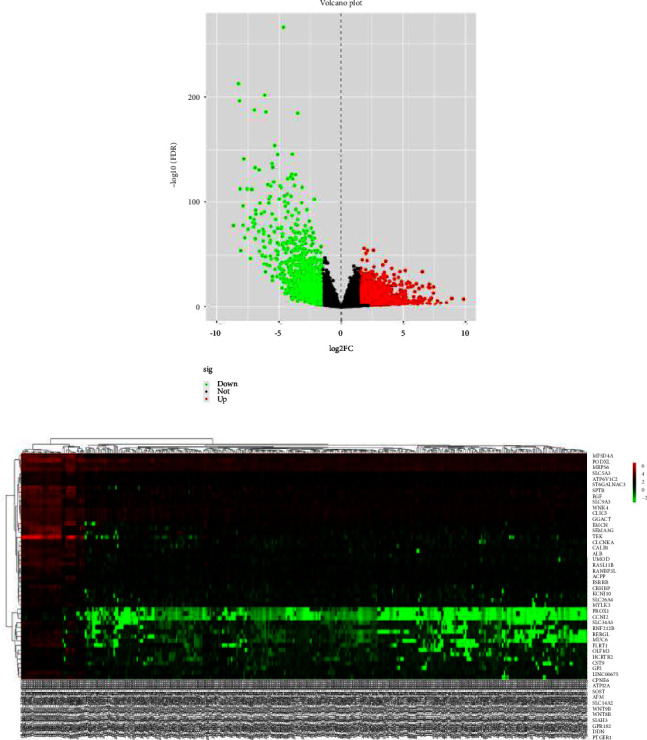
Identification of DEGs in papillary renal cell carcinoma. (a) Volcano plot of the DEGs (|logFC| >2.0 and FDR <0.05 were as the screening conditions). (b) Heatmaps of the top 50 DEGs in papillary renal cell carcinoma and normal kidney tissue. Red indicates that the expression of genes is relatively upregulated, green indicates that the expression of genes is relatively downregulated.

The data for 289 cases of papillary renal cell carcinoma and 32 cases of normal kidney tissue were downloaded from TCGA and used for this study. The data were normalized and logarithmized, probes without corresponding gene annotation information were removed, and repeated probes were removed to finally get the expression profiles of 17,894 genes and 321 samples. Using the edgeR software package, with |logFC|> 2.0 and FDR <0.05 as the screening conditions for differentially expressed genes, a total of 1,992 DEGs were screened for pRCC, including 1,142 upregulated genes and 850 downregulated genes. Using these selected genes, a volcano map (Figure 1) was generated, and the top 50 gene heat maps with the most significant differences were selected (Figure 1(b)).

### 3.2. GO Term and KEGG Pathway Analyses

**Figure 2:**
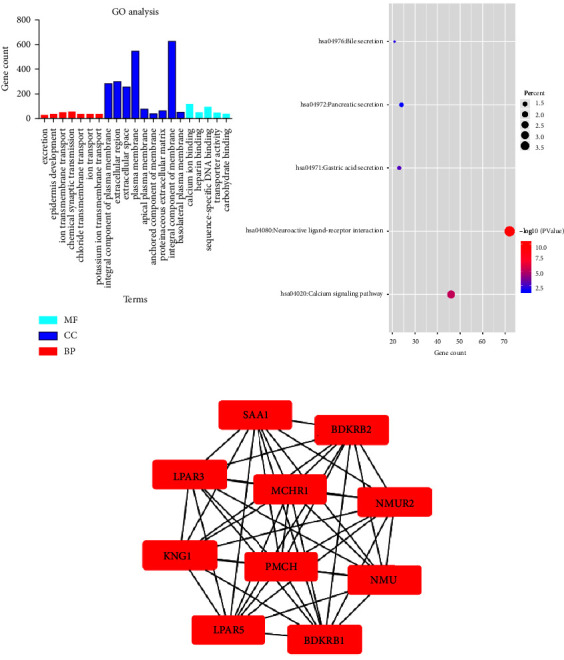
The pathway analyses of DEGs in pRCC. (a) GO enrichment analysis of DEGs in pRCC. GO, Gene Ontology; CC, cellular component; MF, molecular function; BP, biological process. (b) KEGG pathway analysis of DEGs in pRCC. (c) The top 10 hub genes selected from the PPI network.

In order to better understand the relationships between DEGs and pRCC, we input all DEGs into the online tool DAVID to perform GO analysis. The results revealed that, for GO BP analysis, the DEGs of pRCC were mainly enriched in excretion, epidermis development, ion transmembrane transport, chemical synaptic transmission, chloride transmembrane transport, ion transport, and potassium ion transmembrane transport. For GO CC analysis, DEGs were mainly enriched in integral component of plasma membrane, extracellular region, extracellular space, plasma membrane, apical plasma membrane, anchored component of membrane, proteinaceous extracellular matrix, integral component of membrane, and basolateral plasma membrane. For GO analysis, DEGs were mainly enriched in calcium ion binding, heparin binding, sequence-specific DNA binding, transporter activity, and carbohydrate binding. The GO analysis findings are shown in Figure 2 and Table 1.

We next performed KEGG pathway analysis to analyze the pathways at the functional level. The results showed that DEGs were mainly enriched in neuroactive ligand-receptor interaction, calcium signaling pathway, gastric acid secretion, bile secretion, and pancreatic secretion. The KEGG pathways associated with enriched DEGs associated with pRCC are presented in Figure 2(b) and Table 2.

### 3.3. Identification of Hub Genes and Analysis of Modules from PPI Networks

The STRING database was used to construct PPI networks for DEGs related to the pathogenesis of papillary renal cell carcinoma. We used the MCODE in Cytoscape software to obtain the main PPI network (Figure 2(c)), and then used cytoHubba in Cytoscape software to identify the top 10 hub genes in the PPI network (Figure 2(c)): recombinant bradykinin receptor B2 (BDKRB2), neuromodulin U receptor 2 (NMUR2), neuromodulin U (NMU), recombinant bradykinin receptor B1 (BDKRB1), lysophosphatidic acid receptor 5 (LPAR5), Kininogen-1 (KNG1), lysophosphatidic acid receptor 3(LPAR3), serum amyloid A1 (SAA1), melanin-concentrating hormone receptor 1 (MCHR1), and precursor melanin-concentrating hormone (PMCH). These 10 hub genes are presented in Figure 2(c).

### 3.4. Survival Analysis of Hub Genes

**Figure 3:**
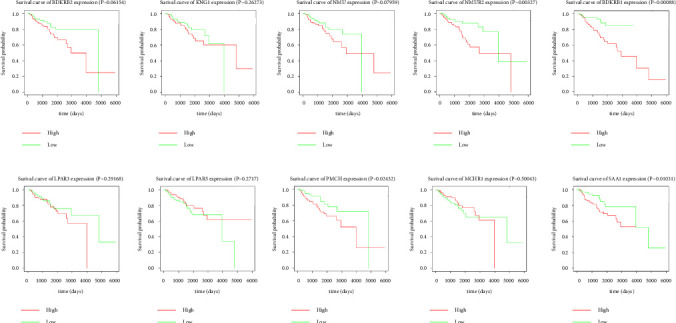
The prognostic values for the 10 hub genes for overall survival of patients with pRCC. (a) Kaplan-Meier plot for BDKRB2, *P*=0.06154; (b) Kaplan-Meier plot for KNG1, *P*=0.26273; (c) Kaplan-Meier plot for NMU, *P*=0.07959; (d) Kaplan-Meier plot for NMUR2, *P*=0.00327; (e) Kaplan-Meier plot for BDKRB1, *P*=0.00088; (f) Kaplan-Meier plot for LPAR3, *P*=0.29168; (g) Kaplan-Meier plot for LPAR5, *P*=0.2717; (h) Kaplan-Meier plot for PMCH, *P*=0.02432; (i) Kaplan-Meier plot for MCHR1, *P*=0.50043; (j) Kaplan-Meier plot for SAA1, *P*=0.01031. A value of *P*  <  0.05 was considered statistically significant.

Expression data for a total of 289 pRCC samples were downloaded from TCGA. The 10 hub genes were grouped by expression levels, and the data were used to conduct survival analyses. Increased expression levels of BDKRB1, NMUR2, PMCH, and SAA1 were associated with a worse survival rate for pRCC patients (Figure 3).

### 3.5. The Expression of Hub Genes BDKRB1, NMUR2, PMCH, and SAA1 in pRCC Tissues and Adjacent Normal Tissues of pRCC Patients

**Figure 4:**
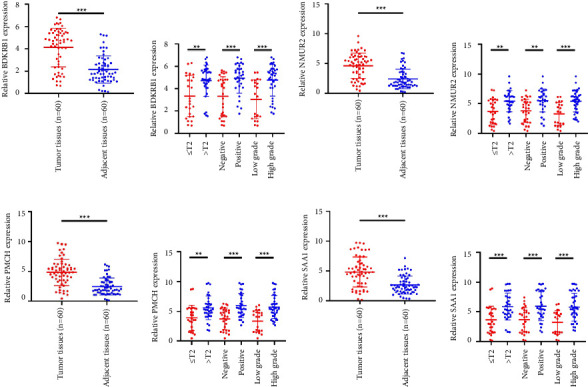
The expression of hub genes BDKRB1 (a), NMUR2 (b), PMCH (c), and SAA1 (d) in pRCC tissues and adjacent normal tissues, as well as the relationship between the expression of hub genes in pRCC tissues and pathological characteristics of pRCC patients (e–h).

We selected 120 tissue samples (including 60 pRCC tissues and 60 normal adjacent tissues) to analyze the expression of hub genes BDKRB1, NMUR2, PMCH, and SAA1 in pRCC tissues by qRT-PCR. The results showed that the expression of hub genes BDKRB1, NMUR2, PMCH, and SAA1 in pRCC tissues was significantly higher than that in the normal adjacent tissues (Figures 4(a), 4(c), 4(e), and 4(g)). To further investigate the correlation between hub genes BDKRB1, NMUR2, PMCH, and SAA1 expression and pathological features of pRCC, the above samples were divided into high (above the mean) and low (below the mean) hub genes expression groups. Subsequently, the Chi-square test was used to analyze the relationship between hub genes BDKRB1, NMUR2, PMCH, and SAA1 expression level and pathological characteristics of pRCC patients, and the results showed that the expression level of hub genes BDKRB1, NMUR2, PMCH, and SAA1 expression in pRCC tissues were significantly positively correlated with tumor stage, lymph node metastasis, and histopathological grade of pRCC patients (Figures 4(b), 4(d), 4(f), and 4(h)), while the relationship with gender and age of patients was not statistically significant (Tables 3–6).

### 3.6. Relationship between Hub Genes BDKRB1, NMUR2, PMCH, and SAA1 Expression and Prognosis of Patients with pRCC

**Figure 5:**
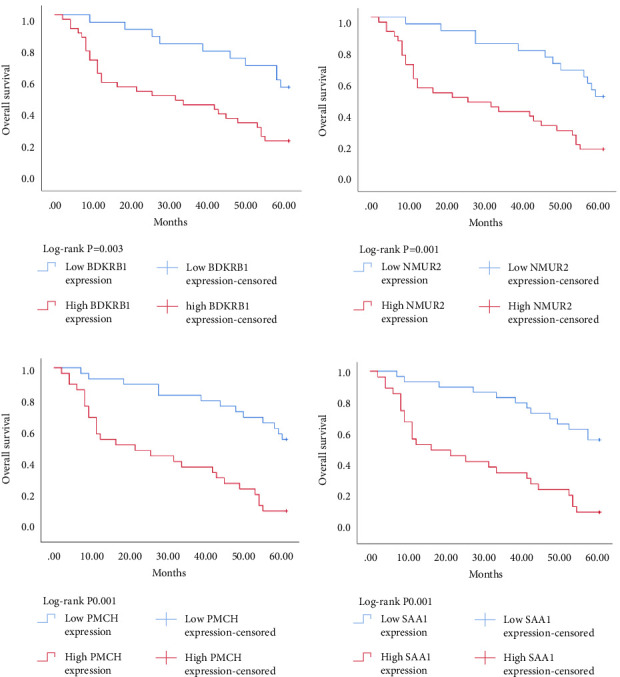
Relationship between hub genes BDKRB1 (a), NMUR2 (b), PMCH (c), and SAA1 (d) expression and prognosis of patients with pRCC.

The Kaplan–Meier survival analysis was used to study the relationship between hub genes BDKRB1, NMUR2, PMCH, and SAA1 expression and prognosis of patients with pRCC. The results showed that the overall survival rate of patients with high hub genes BDKRB1, NMUR2, PMCH, and SAA1 expression was significantly lower than that of patients with low hub genes BDKRB1, NMUR2, PMCH, and SAA1 expression (Figure 5). Then we conducted the COX proportional risk model analysis. The univariate and multivariate analyses showed that the expression of hub genes BDKRB1, NMUR2, PMCH, and SAA1 were independent risk factor for prognosis in patients with pRCC (Tables 7–10).

## 4. Discussion

Most patients with pRCC have no obvious symptoms or signs at the time of diagnosis, but the disease is often found by B-ultrasound or CT examination during a physical examination. Very few patients exhibit the typical triad signs of kidney cancer: hematuria, abdominal mass, and lumbar pain, and the patients that do exhibit these signs typically have advanced disease. The overall prognosis of pRCC is better than that of ccRCC, but pRCC prognosis is significantly worse than that of ccRCC when pRCC invades the renal vein and/or the inferior vena cava [10]. There is currently no specific treatment for pRCC, and surgical treatment is the first choice in clinical practice. The prognosis of advanced patients is poor, a pRCC is insensitive to radiotherapy and chemotherapy. Therefore, the study of the mechanisms of pRCC development and metastasis will help improve clinical diagnosis and treatment.

In this study, bioinformatics technology was used to mine pRCC transcriptomic data downloaded from TCGA. A total of 1,992 DEGs were identified, including 1,142 upregulated genes and 850 downregulated genes. We performed GO and KEGG pathway enrichment analyses to explore interactions between DEGs. The GO analysis revealed that 1,992 DEGs were significantly enriched in 21 terms, including excretion, epidermis development, ion transmembrane transport, chemical synaptic transmission, chloride transmembrane transport, ion transport, potassium ion transmembrane transport, integral component of plasma membrane, extracellular region, extracellular space, plasma membrane, apical plasma membrane, anchored component of membrane, proteinaceous extracellular matrix, integral component of membrane, basolateral plasma membrane, calcium ion binding, heparin binding, sequence-specific DNA binding, transporter activity, and carbohydrate binding. In addition, the KEGG pathway analysis revealed that 1,992 DEGs were significantly enriched in five pathways, including neuroactive ligand-receptor interaction, calcium signaling pathway, gastric acid secretion, bile secretion, and pancreatic secretion. According to the STRING results, we constructed the PPI network. Then hub genes were selected with a high degree of interaction in the PPI network, including BDKRB2, NMUR2, NMU, BDKRB1, LPAR5, KNG1, LPAR3, SAA1, MCHR1, and PMCH. Further analysis of survival related to the expression of these hub genes revealed that BDKRB1, NMUR2, PMCH, and SAA1 are the key genes for the development of pRCC.

One hub gene, BDKRB1, is a well-established tumor suppressor gene, which is frequently mutated in familial breast and ovarian cancers. The gene product of BDKRB1 functions in a number of cellular pathways that maintain genomic stability, including DNA damage-induced cell cycle checkpoint activation, DNA damage repair, protein ubiquitination, chromatin remodeling, as well as transcriptional regulation and apoptosis. In this study, we found the role of BRCA1 in tumor suppression and DNA damage response, including DNA damage-induced cell cycle checkpoint activation and DNA damage repair. The other hub gene KNG1 (Kininogen-1) is expressed at low level in glioma cells. KNG1 can exert antiangiogenic properties and inhibit the proliferation of endothelial cells [11]. Previous work showed that KNG1 can be used as a serum biomarker for colorectal cancer [12]. Overexpression of the KNG1 inhibited proliferation and induces apoptosis of glioma cells [11]. In this study, KNG1 expression was downregulated in pRCC, which may be associated with the viability and angiogenesis of pRCC, but the analysis revealed no statistical impact of expression of this gene on survival, suggesting further investigation into the relationship between this gene and pRCC is required. Lysophosphatidic acid (LPA) is an extracellular biological lipid that interacts with G protein-coupled LPA receptors (LPAR1 to LPAR6) [13]. The lysophosphatidic acid receptor-3 (LPAR3) mediates viability among malignant cells and aggressiveness among certain tumors [14]. LPAR3 has been characterized as the major promoter of long-term viability in melanoma cells [15]. Other studies found that increased expression of LPAR3 increases malignancy in breast and ovarian cancers in vivo [16, 17]. In this study, LPAR3 was identified as a downregulated gene in pRCC. It was reported with the involvement of LPA5 in the activation of tumor progression in pancreatic cancer cells [13]. Bradykinin (BK) is produced in the inflammatory tissue microenvironment, where it acts in cell proliferation, leukocyte activation, cell migration, and endothelial cell activation [18]. BDKRB1 and BDKRB2 belong to the rhodopsin family of G protein-coupled receptors. The activation of BDKRB1 leads to the activation of macrophages, dendritic cells, and other cells in the tumor microenvironment, which have angiogenic properties and is related to the proliferation of malignant tumors [19]. BDKRB1 contributes to interleukin-8 production and glioblastoma migration [20]. Wang et al. reported that inhibition of BDKRB2, but not the B1 receptor, attenuated bradykinin-mediated invasion and migration in colorectal cancer cells and inhibited ERK1/2 activation and IL-6 production [21]. Thus, the identification of inhibitors against BDKRB1 may be a reasonable strategy to suppress pRCC. Neuromodulin U (NMU) activates the G protein-coupled receptor NMUR2, and NMU signaling interacts with several cancer-related pathways, including the WNT receptor cascade, resulting in increased activation of WNT/planar cell polarity (PCP) effector RAC1, which promotes tumor cell invasion and metastasis [22]. NMUR2 is a receptor that enhances NMU-mediated cell motility and invasion in human pancreas and endometrial cancer cells [23, 24]. Hub genes NMU and NMUR2 have not previously been reported to play roles in pRCC. PMCH encodes the 165 aa prohormone promelanin-concentrating hormone (PMCH), which is proteolytically processed into several peptides, including the oncogenic peptide melanin-concentrating hormone (MCH) [25]. In this study we found that increased expression of PMCH was associated with poor survival in patients with pRCC, suggesting PMCH may be a potential diagnostic biomarker or predictor of prognosis. Human serum amyloid A (SAA) is a high-density lipoprotein (HDL)-related lipoprotein with major roles in the regulation of inflammation and cholesterol transport [26]. Human serum amyloid A (SAA) has been widely regarded as an accurate and sensitive indicator of inflammation, which can be synthesized by the liver and cancer cells [27]. SAA1 regulates cell adhesion and migration and binding to laminin by inducing cytokine expression [28]. A previous study reported a relationship between increased SAA1 concentration and poor prognosis and distant metastasis in ccRCC patients [29].

In conclusion, bioinformatics analysis was used to identify DEGs that may be involved in the development or progression of the pRCC. This study identified several genes that may be involved in the pathology of papillary renal cell carcinoma. BDKRB1, NMUR2, PMCH, and SAA1 may contribute to the occurrence and development of papillary renal cell carcinoma. This identification of specific biological functions that may be involved in the mechanism of pRCC development provides new clues and directions for efforts to develop future treatments for papillary renal cell carcinoma.

## Figures and Tables

**Table 1:** Gene ontology analysis of DEGs associated with pRCC.

Category	Term	Description	Count	*P* value
BP	GO:0007588	Excretion	18	4.00*E* − 05
BP	GO:0008544	Epidermis development	27	2.31*E* − 04
BP	GO:0034220	Ion transmembrane transport	47	4.69*E* − 04
BP	GO:0007268	Chemical synaptic transmission	51	7.34*E* − 04
BP	GO:1902476	Chloride transmembrane transport	26	0.005783318
BP	GO:0006811	Ion transport	31	0.011414198
BP	GO:0071805	Potassium ion transmembrane transport	29	0.034687627
CC	GO:0005887	Integral component of plasma membrane	279	2.76*E* − 27
CC	GO:0005576	Extracellular region	293	8.03*E* − 23
CC	GO:0005615	Extracellular space	252	8.22*E* − 21
CC	GO:0005886	Plasma membrane	543	1.13*E* − 10
CC	GO:0016324	Apical plasma membrane	69	2.70*E* − 08
CC	GO:0031225	Anchored component of membrane	37	2.04e − 07
CC	GO:0005578	Proteinaceous extracellular matrix	61	2.45*E* − 06
CC	GO:0016021	Integral component of membrane	620	3.43*E* − 05
CC	GO:0016323	Basolateral plasma membrane	44	6.86*E* − 05
MF	GO:0005509	Calcium ion binding	119	8.07*E* − 06
MF	GO:0008201	Heparin binding	41	2.33*E* − 05
MF	GO:0043565	Sequence-specific DNA binding	89	1.82*E* − 04
MF	GO:0005215	Transporter activity	43	0.002430787
MF	GO:0030246	Carbohydrate binding	40	0.017062891

**Table 2:** KEGG pathway analysis of DEGs associated with pRCC.

Category	Term	Description	Count	*P* value
KEGG	hsa04080	Neuroactive ligand-receptor interaction	72	4.38*E* − 11
KEGG	hsa04020	Calcium signaling pathway	46	5.31*E* − 06
KEGG	hsa04971	Gastric acid secretion	23	0.002127995
KEGG	hsa04976	Bile secretion	21	0.012213919
KEGG	hsa04972	Pancreatic secretion	24	0.046905158

**Table 3:** The relationship between BDKRB1 expression level in pRCC and pathology features of pRCC patients (*n* = 60).

Characteristics	BDKRB1		Chi-squared test	*P* value
	Low no. cases	High no. cases		
All patients	(*n* = 23)	(*n* = 37)		
Gender			0.035	0.852
Male	13	20		
Female	10	17		
Age (years)			0.012	0.914
≤60	9	15		
>60	14	22		
Tumor stage			7.274	0.007
≤T2	15	11		
>T2	8	26		
Lymph-node metastasis			5.711	0.017
Negative	16	14		
Positive	7	23		
Pathology grade			6.332	0.012
Low grade	13	9		
High grade	10	28		

**Table 4:** The relationship between NMUR2 expression level in pRCC and pathology features of pRCC patients (*n* = 60).

Characteristics	NMUR2		Chi-squared test	*P* value
	Low no. cases	High no. cases		
All patients	(*n* = 25)	(*n* = 35)		
Gender			1.009	0.315
Male	14	15		
Female	11	20		
Age (years)			0.156	0.693
≤60	12	15		
>60	13	20		
Tumor stage			6.251	0.012
≤T2	16	11		
>T2	9	24		
Lymph-node metastasis			7.096	0.008
Negative	18	13		
Positive	7	22		
Pathology grade			6.898	0.009
Low grade	14	8		
High grade	11	27		

**Table 5:** The relationship between PMCH expression level in pRCC and pathology features of pRCC patients (*n* = 60).

Characteristics	PMCH		Chi-squared test	*P* value
	Low no. cases	High no. cases		
All patients	(*n* = 30)	(*n* = 30)		
Gender			0.067	0.795
Male	17	16		
Female	13	14		
Age (years)			0.278	0.598
≤60	13	11		
>60	17	19		
Tumor stage			4.344	0.037
≤T2	17	9		
>T2	13	21		
Lymph-node metastasis			6.667	0.01
Negative	20	10		
Positive	10	20		
Pathology grade			7.177	0.007
Low grade	16	6		
High grade	14	24		

**Table 6:** The relationship between SAA1 expression level in pRCC and pathology features of pRCC patients (*n* = 60).

Characteristics	SAA1		Chi-squared test	*P* value
	Low no. cases	High no. cases		
All patients	(*n* = 31)	(*n* = 29)		
Gender			0.012	0.913
Male	17	15		
Female	15	14		
Age (years)			0.63	0.427
≤60	15	17		
>60	16	12		
Tumor stage			8.21	0.004
≤T2	20	8		
>T2	11	21		
Lymph-node metastasis			4.312	0.038
Negative	19	10		
Positive	12	19		
Pathology grade			9.121	0.003
Low grade	17	5		
High grade	14	24		

**Table 7:** Univariate and multivariate analysis of overall survival in patients with pRCC (*n* = 60).

Variable for overall survival	Univariate analysis			Multivariate analysis		
	HR	95% CI	*P*	HR	95% CI	*P*
Gender			0.108			
Male vs. female	0.581	0.3–1.126				
Ages (years)			0.134			
≤60 vs. >60	1.659	0.855–3.218				
Pathology grade			0.403			
Low grade vs. high grade	1.331	0.681–2.603				
Tumor stage			0.03			0.716
≤T2 vs. >T2	2.11	1.076–4.137		1.616	0.806–3.239	
Lymph-node metastasis			0.012			0.111
Negative vs. positive	2.31	1.2–4.448		1.743	0.880–3.450	
BDKRB1 expression			0.005			0.065
Low vs. high	2.829	1.366–5.858		2.082	0.957–4.532	

Abbreviations: HR, hazard ratio; CI, confidence interval.

**Table 8:** Univariate and multivariate analysis of overall survival in patients with pRCC (*n* = 60).

Variable for overall survival	Univariate analysis			Multivariate analysis		
	HR	95% CI	*P*	HR	95% CI	*P*
Gender			0.727			
Male vs. female	1.118	0.598–2.089				
Ages (years)			0.308			
≤60 vs. >60	1.386	0.74–2.598				
Pathology grade			0.489			
Low grade vs. high grade	1.254	0.661–2.38				
Tumor stage			0.007			0.086
≤T2 vs. >T2	2.461	1.278–4.739		0.548	0.276–1.089	
Lymph-node metastasis			0.012			0.209
Negative vs. positive	2.252	1.198–4.234		0.652	0.335–1.270	
NMUR2 expression			0.002			0.021
Low vs. high	2.95	1.488–5.847		0.432	0.212–0.882	

Abbreviations: HR, hazard ratio; CI, confidence interval.

**Table 9:** Univariate and multivariate analysis of overall survival in patients with pRCC (*n* = 60).

Variable for overall survival	Univariate analysis			Multivariate analysis		
	HR	95% CI	*P*	HR	95% CI	*P*
Gender			0.16			
Male vs. female	0.629	0.329–1.201				
Ages (years)			0.107			
≤60 vs. >60	1.716	0.89–3.31				
Pathology grade			0.49			
Low grade vs. high grade	1.259	0.654–2.423				
Tumor stage			0.131			
≤T2 vs. >T2	0.608	0.318–1.16				
Lymph-node metastasis			0.02			0.373
Negative vs. positive	0.468	0.246–0.889		0.732	0.368–1.456	
PMCH expression			0			0.001
Low vs. high	0.256	0.13–0.507		0.289	0.139–0.601	

Abbreviations: HR, hazard ratio; CI, confidence interval.

**Table 10:** Univariate and multivariate analysis of overall survival in patients with pRCC (*n* = 60).

Variable for overall survival	Univariate analysis			Multivariate analysis		
	HR	95% CI	*P*	HR	95% CI	*P*
Gender			0.381			
Male vs. female	0.75	0.393–1.429				
Ages (years)			0.572			
≤60 vs. >60	1.202	0.635–2.275				
Pathology grade						
Low grade vs. high grade	1.335	0.683–2.609	0.399			
Tumor stage			0.016			0.209
≤T2 vs. >T2	0.443	0.228–0.861		0.639	0.318–1.284	
Lymph-node metastasis			0.036			0.333
Negative vs. positive	0.497	0.259–0.956		0.714	0.361–1.413	
SAA1 expression			0			0.004
Low vs. high	0.261	0.132–0.516		0.332	0.158–0.7	

Abbreviations: HR, hazard ratio; CI, confidence interval.

## Data Availability

All data generated or analyzed during this study are included within this article.
